# Climate change contribution to the 2023 autumn temperature records in Vienna

**DOI:** 10.1038/s41598-024-54822-2

**Published:** 2024-02-20

**Authors:** Johannes Laimighofer, Herbert Formayer

**Affiliations:** 1 Institute of Meteorology and Climatology, Department of Water, Atmosphere and Environment, University of Natural Resources and Life Sciences, Vienna, Austria; 2 Institute of Statistics, Department of Landscape, Spatial and Infrastructure Sciences, University of Natural Resources and Life Sciences, Vienna, Austria

**Keywords:** Climate change, Natural hazards

## Abstract

Global monthly mean temperature continuously broke records in the year 2023 since June till October. This also happened widespread at September and October in Austria, but monthly temperature records on a local scale, such as in the mid latitudes like Austria, show less persistence than global or continental averages. This makes the autumn temperature extremes in Vienna (Austria) even more striking. Considering the compound occurrence of such an event at actual climate results in a return period of 324 years, which makes it extraordinary itself. Considering climate change, the compound event of two consecutive extreme high temperature records in autumn 2023 yields return periods of about 10,000 years until the second half of the twentieth century, which partly exceeds the length of the Holocene. Focusing on moderate compound extremes of the last 10 years (2014–2023), these reach return periods of 100 years up to 1960, but are now likely to happen every 15 years. Compound extremes in summer (July and August) present a higher decrease of the return period in Vienna over the last 250 years, possible leading to even more severe impacts on ecosystems and society.

## Introduction

In Austria the first two months of the autumn 2023 recorded extreme positive anomalies of monthly temperature. The September and October set new temperature records at most meteorological stations in Austria. The occurrence of two consecutive months with maximum temperature records in Austria, did not get much attention from the general public, as this also happened for the global mean temperature^1^ and even for the European mean temperature. But there are significant differences between large scale spatial averages like continental or even global mean temperatures^2,3^ and individual local station observations^4^.

Global temperature exceeded existing temperature records in 2023 from June to October and all other months were ranked in the top seven records for each month (Supplementary Fig. 1). Persistence of consecutive temperature records is however lower in local climates^5,6^. Temperature records in Vienna for example paint a different picture. Although July 2023 is the 3rd highest recorded anomaly from 1850 to 2023 in Vienna, only January is ranked under the top ten anomalies in 2023. April and May can be considered even average or below average months for this period (Supplementary Fig. 1). Nevertheless, September and October exhibit two consecutive temperature records widespread in Austria. For Vienna the October 2023 is the second highest anomaly, but only because the time series reaches back to the eighteenth century and includes the slightly warmer October of 1811. These two diverging pictures of global and local temperature, emphasize the extraordinary temperature extreme in autumn in Vienna 2023.

One of the explanations for the current high global temperatures is the ongoing El Nino event. Impact of El Nino on the temperature and precipitation pattern in Europe is widely discussed^7,8^ and the possible effect for the climate in Vienna has to be specifically addressed here. An own statistical test (see Supplementary material) indicate a negligible influence on the Austrian temperature anomaly in September and October.

The main objectives of this study are threefold: (i) to evaluate the occurrence probability of the compound extreme autumn temperature 2023 in Vienna in a world without anthropogenic climate change, (ii) to quantify the occurrence probability of autumn 2023 in the real world with climate change, and (iii) to assess the return period of an average compound event of the last ten years for autumn and summer in Vienna. All the analysis are based on a 249 year long record of mean monthly temperature.

## Results

### Univariate extreme events

Average monthly temperature in Vienna in the pre-industrial period (before 1900) were 14.9 ^∘^C in September and 9.6 ^∘^C in October. Autumn temperature in Vienna did slightly decrease to 14.5 ^∘^C in September and 9.3 in October between 1901 and 1960. Since then, the temperature increased significantly (tested by a Welch’s t-test^9^ on an alpha level of 0.01) to 15.7° in September and 10.5 in October for the current climate reference period of 1991 to 2020.

**Figure 1:**
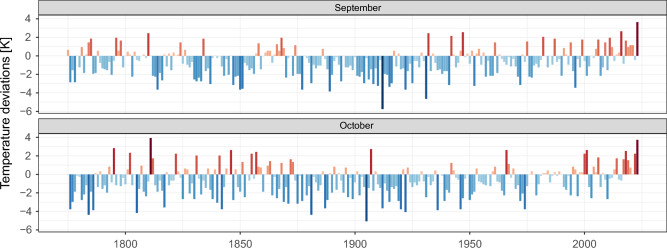
Monthly deviations. Visualization of the monthly deviations for Vienna Hohe-Warte for the full observational period (1775–2023). Deviations are calculated by subtracting the climate normal period (1991–2020) from the absolute values.

Figure 1 shows the monthly anomalies for Vienna in reference to the current climate period of 1991–2020. One can see somewhat higher temperatures in the pre-industrial period, compared to a colder period at the end of the nineteenth century to the beginning of the twentieth century. The effect of anthropogenic climate change can best be spotted since the start of the twenty first century, where only 39% (43%) of the September (October) values have a deviation below zero, in contrast to 71% (70%), for the time before 2000. The highest anomaly for September occurred in 2023 with 3.65 K over the climatic reference period, whereas the second highest anomaly in 2016 yields only a surplus of 2.65 K. In October the first two extreme exceed all other anomalies by almost 1 K. The highest anomaly was in 1811 with 3.93 K, followed by the extreme year of 2023 with a positive anomaly of 3.73 K.

Description of extreme events often appears in return periods. In a non-stationary world of climate change, where the average temperature is increasing, direct computation of return periods would be misleading. Therefore, the trend of the monthly deviations is removed by a Loess-model (for each month separately) and the residuals are checked for auto-correlation and a remaining trend. Both test results are not significant for September and October. The resulting residuals are then used to compute return periods, which can be compared over the full time series. Our main interest are the exceeding probability of the residuals for each single month. Considering return periods larger than 100 years (occurrence probability of 0.01), two events for September, and three events for October can be identified. In September the residual of 1932 yields a return period of 215 years and the year 1947 obtains a return period of 195 years. An extraordinary event happened in October 1811 with a return period of 841 years. Other outstanding events in October are the year 1907 (return period of 209 years) and 1795 (160 years). What is striking, is that the monthly residuals of 2023 have only a return period of 53 years in September and of 30 years in October. This highlights that these events were not that extreme on its own. Note, that these return periods are calculated on the residuals, meaning they are conditional to the specific climate period—so in a world without climate change. The actual anomalies of 2023 would be much more unlikely to occur in the past. This can be observed from the event of 1811, which is similar to the anomaly of October 2023. In 1811 this yields a return period of 841 years, whereas in our current climate we expect such events to happen every 30 years.

### Compound extreme events

The first analysis focused on the individual occurrence probability of each month. In a next step, we analyze the probability of a compound event in September and October. We assume that the temperature deviations in September and October are not independent, this entails that higher anomalies in September lead to higher anomalies in October and vice versa. This assumption is checked by a test for independence^10^ between the two months, which is significant on an alpha level of 0.05 (*p*-value of 0.013). The relationship between September and October monthly temperature is then approximated by a (Gumbel-) copula model, which can model the joint dependence and gives us the opportunity to estimate the joint probability of the 2023 autumn temperature records in Vienna. The strength of the correlation between the two months can be quantified by Kendalls^11^ $\tau$-value, which is 0.11, indicating a weak relationship (0 means independence, 1 is a perfect correlation) of monthly temperature deviations in September and October.

**Figure 2:**
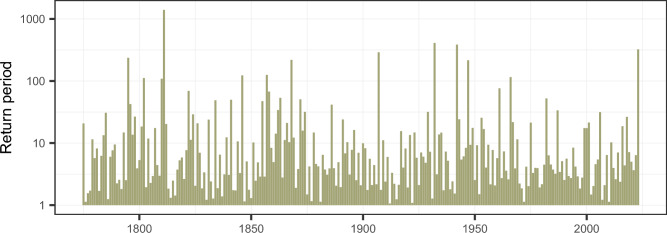
Compound extremes. Return periods (in years) of the compound effect of September and October temperature anomalies. The y-axis is presented on a log-scale.

Regarding the single events of 2023, it is already retained, that they were not exceptional itself in a world without climate change. This analysis is now extended to the compound effect of autumn temperature records. Figure 2 visualizes the return periods of these compound events, computed for every year. High return periods (low occurrence probabilities) indicate that both events—September and October—have a high univariate return period in the same year. The most extreme event is the year 1811, where October had a return period of 841 years and the compound event has a return period of 1400 years (90% confidence interval 1039–2753 years). Severe compound events can also be found in 1932 (return period of 411 years) and 1942 (386 years). 2023 is ranked fourth under the compound extremes with a return period of 324 years (90% confidence interval 176–1562 years). This highlights that the year 2023, would be an extreme event in any of the last 249 years, as the return period was calculated in respect to a de-trended time series. Nevertheless, the year 2023 was not the most extreme, as other years were even more unlikely. This points out that the exceptional event of 2023, was not driven by the persistence of the current global temperature extremes, and worse compound extremes would have been possible.

**Figure 3:**
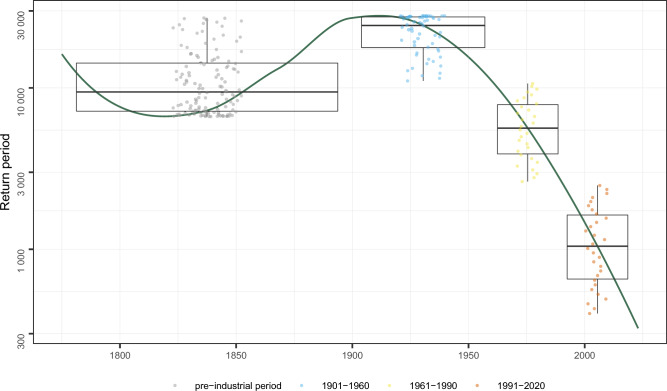
Return period extreme event 2023. The boxplots show the distribution for four different climate periods. The corresponding points are the individual years, randomly ordered over the x-axis. The line presents the temporal process. The y-axis is presented on a log-scale.

Up to now we only considered a world without climate change. To quantify the impact of climate change, we quantify the occurrence probability of autumn 2023 temperature records for the last 249 years. Therefore, the difference between the anomalies of the actual event and the fitted trend is calculated. Each pair of residuals (September and October) is then used to estimate the corresponding return period. Figure 3 presents the results for each year and the distribution for different climatic reference periods. An extreme event as in the year 2023 was almost impossible until the second half of the twentieth century where the average return periods are larger than 10,000 years and partly exceed the length of the Holocene. The return period drops below 10,000 years for the climate period from 1961–1990. Occurrence probability that make such an event possible only appear in the twenty first century, where the return period decreases to about 750 years. This highlights, that although the event of 2023 itself was not the most extreme for the last 249 years when considering the climate of the respective period, the appearance of such an event, was only possible due to climate change.

**Figure 4:**
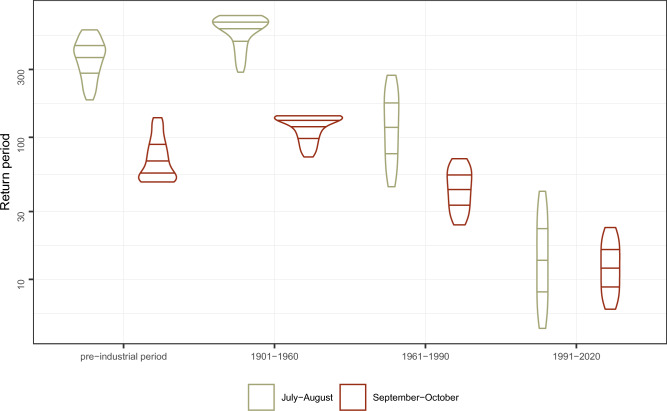
Comparison of compound events. Violin plots and 25%, 50% and 50% quantiles for the compound return periods for two average months of the period from 2014-2023. Analysis is performed for the compound events of September-October and July-August.

The preceding analysis showed that the compound event of 2023 was only possible due to the increasing temperature induced by anthropogenic climate change. Nevertheless, more common events may give better insights to the change in the likelihood of these compound extreme events. For this the probability for a joint event for September and October was considered, that corresponds to the average of the last ten years (2014-2023) for each individual time series. This analysis was not only performed for the months of September and October, but additionally for a compound extreme event in July and August (Fig. 4).

In the pre-industrial period, the return period for September and October was on average 73 years, but increased for the first half of the twentieth century to almost 120 years. It then sharply drops to an average of 13 years for the period from 1991–2020. A similar functional relationship can be identified for the compound extreme event of July and August, where the return period for the pre-industrial area would be 372 years, and declines till 1991–2020 to 17 years. This means that an event, that is currently possible every 17 years, and probably more frequent in the future, had a return period of more than 350 years until 1960. This comparison of the two consecutive months July and August highlights, that the behavior of the actual record months September and October is not an artifact of the season.

## Discussion

In this work we investigate the probability of occurrence of two consecutive months with record high temperature, as it took place widely in September and October 2023 in Austria. Others than for the global mean temperature, where the persistence of ocean temperature anomalies can last several months and though the persistence of monthly anomalies is quite high, monthly temperature anomalies in Austria, and general in the mid latitudes^3^, have a low persistence. This is because the length of periods with the same temperature anomaly (e.g. cold spells or heat waves) is in the order of several days up to two or three weeks. Also El Nino, that is contributing to the current monthly global mean temperature record, has no significant impact on the persistence of weather pattern in Vienna, as shown in the supplementary material.

To quantify return periods of these events in Austria, we used the data from the station Vienna Hohe-Warte, with homogenized monthly temperature observations since 1775. Based on the actual climate, defined as the period 1991–2020, the temperature anomaly for September 2023 has a return period of 53 years and 30 years in October. Related to the average climate at their occurrence, the September 1932 with a return period of 215 years and the October 1811, with a return period of 841 years, have been considerably more extreme. Given that the October 1811 was only 0,2 ^∘^C warmer than 2023, the different return period highlight the impact of anthropogenic climate change on the occurrence of the individual monthly anomalies.

The compound event of two consecutive extreme positive anomalies as September and October 2023 in Vienna, based on the actual climate of 1991 to 2020, has a return period of 349 years. This is clearly a rare event, but compared to the de-trended time series, it is only the forth highest extreme event in the 249 year time series of Vienna. If the absolute temperature anomalies are considered, then the impact of anthropogenic climate change becomes visible: Before the middle of the twentieth century, the return periods increases to 10.000 years or even higher, exceeding the length of the Holocene. Only after the middle of the twenty first century, the return period drops below 10.000 year. This illustrates what an extraordinary event the sequence of the months September and October 2023 in Vienna was, and that this was only made possible by anthropogenic climate change.

We additionally investigated the probability of occurrence for the consecutive temperature anomalies for the months July and August. The dependency structure between temperature records in July and August (Kendall $\tau$ of 0.2) is stronger than for September and October ($\tau = 0.11$). Considering moderate temperature anomalies of the last 10 years for the summer months, the return period increases by a factor of 20 between the actual climate 1991–2020 and the time before the middle of the twentieth century. This is even more striking when considering the compound event of 2023 for the summer months (Supplementary Fig. 3), which is currently likely to occur every 6 years, but return periods before 1960 were over 750 years for such an event. Of course, the occurrence of two month with extreme positive temperature deviations during the warmest phase of the year, would lead to extreme heat stress. Monthly mean temperatures are highly correlated with the occurrence of days with high temperatures^12^ and heat waves. Hot months in summer with associated heat stress^13^, have the potential to increase the mortality in Vienna significantly^14^. Therefore the occurrence of such an event in July and August would have much more serious effects in Austria than the current one in autumn.

## Methods

### Data

This study uses three different datasets and all of them are freely accessible and available to download. The long-term record of the station Vienna Hohe-Warte—which is the main data source—is provided by GeoSphere Austria over the HISALP dataset^15^. The monthly mean temperature series has no missing values^15^ and the single time series ranges from 1775 to 2023. The current download (2023-09-11) includes no values for September and October, which where provided by GeoSphere Austria through personal communication. The station data was homogenized (not by the authors), by using the serial auto-correlation between neighbouring stations^16,17^. Therefore, a sub-region with a lower correlation-threshold of 0.85 between each station^15^ is build. For this sub-region a mean station density of 400 km—as reported for the HISTALP dataset in the eighteenth century—is sufficient for homogenization of mean monthly temperature^16^. The homogenization procedure additionally includes visualization of the spatial coherence of monthly air temperature and extensive use of the metadata, to detect outliers and avoid unreliable anomalies^15^.

For visualization of the global mean temperature the HadCRUT.5.0.2.0^18^ is used. The global temperature anomalies are based on an ensemble average of 200 regional ensemble realizations and are available for a period from 1850 to 2023. Finally, for estimating the impact of the current strong El Nino event, the multivariate ENSO index^19^ was downloaded at NOAA. The monthly updated ENSO index ranges from 1979 to 2022.

### Statistical analysis

Data analysis in this study was performed in R^20^, by additional use of the following packages: tidyverse^21^, trend^22^, RColorBrewer^23^, zoo^24^, wesanderson^25^, lmtest^26^, and gridExtra^27^.

#### De-trending of the monthly time series

First, monthly anomalies are calculated out of the absolute monthly mean temperature series, by subtracting the mean of the climate normal period 1991–2020—which is recommended by the World Meteorological Organization (WMO^28^)—for each month separately. Note, that except for Fig. 4, Supplementary Fig. 1 and Supplementary Fig. 3 the months of September and October are the main interest of this study. The resulting anomalies are then used to fit a Loess-model^29^ by least squares, to account for the inter-annual variability and a possible increase in temperature due to climate change. The solely predictor variable included in the Loess-model is the numeric representation of the years. The produced fit should only give a smooth approximation to the data (an example of the fit is given in Supplementary Fig. 2). This de-trending procedure is performed for every month separately and the residuals of the models should not be auto-correlated or include any trend. This task is carried out to yield a time series that is stationary and can be used for fitting a univariate, or bivariate distribution^30^. Hence, the final residuals are checked for auto-correlation by the Durbin-Watson test^31^, and the Mann–Kendall trend test^32^ is applied to test if the trend was removed. Both tests are performed on a significance level of 0.05.

#### Modeling univariate extreme events

The temperature residuals of September ($sep_{yr}$) and October ($oct_{yr}$) are then used as an input for fitting a univariate distribution. Each month is fitted to a normal distribution, as we assume normal distributed residuals. Mean and standard deviation of the normal distribution are estimated by L-moments^33–35^, which should give more robust estimates. For each of the residuals the exceedance probability and the corresponding return period is computed by:

1 $$\begin{aligned} RP_{sep_{yr}} = \frac{1}{1 - F_{sep}(sep_{yr})}, \end{aligned}$$

where $RP_{sep_{yr}}$ for example is the return period of a specific year for the residuals in September ($sep_{yr}$) and $F_{sep}$ is the cumulative distribution function of the estimated normal distribution for September. This can be equally written for the months of October, July and August.

#### Modeling compound extreme events

The main interest of this study is the concurrent occurrence of high extremes in September and October in the local climate of Vienna. The persistence of maximum anomalies is higher in a global climatology, than for local conditions. Nevertheless, the dependency between two consecutive months can be approached by the use of copulas^36^, which jointly model the marginal distributions of two univariate distributions. Copulas are widely used in climate research for estimating the joint probability of two dependent events^30^. Mainly, if there are two univariate continuous and independent distributions, as $F_{sep}$ and $F_{oct}$, then any multivariate cumulative distribution function can be defined as a copula model with $C(F_{sep}, F_{oct})$, and respectively also for July and August. This study is focusing on exceeding probabilities (positive extremes), where a suitable approach is to use Archimedean copulas^10,37^. A special case of the Archimedean copula is the Gumbel copula^38^, which gives more emphasis on the upper tails of the joint distribution, and seems an appropriate approximation in our case. The Gumbel copula (for September and October) can be written as:

2 $$\begin{aligned} C = exp [-((-log(F_{sep}(sep_{yr}))^\theta + (-log(F_{oct}(oct_{yr}))^\theta )^{1/\theta }], \end{aligned}$$

where $\theta$ is the parameter modeling the dependence structure between the September and October. A value of $\theta = 1$ would indicate total independence between the two months, and larger values relate to a stronger relationship. $\theta$ is fitted by maximum likelihood^39^ in the VineCopula package in R^40^.

Uncertainty of the $\theta$ parameter is assessed by bootstrapping a 1000-times over the de-trended residuals of each month. The 0.05, 0.5 and 0.95 quantiles are then used for estimating the final model, where the 0.05 and 0.95 quantiles of $\theta$ produce a 90% confidence interval (the upper parameter interval produces the lower interval of the return period), and the 0.5 quantile gives the model for the point estimate used in the study.

As for the evaluation of the univariate time series, a return period (of exceedance probability) is estimated for the compound event. In case of copulas one can differ between the return period of an *OR* event, or an *AND* event^41^. An *OR* event describes the probability that either September or October is above a specific pair of quantiles ($q_{sep}, q_{oct}$). An *AND* event is determined by the probability that both months—September and October (or July and August)—exceed their specific quantiles $q_{sep}$ and $q_{oct}$. This study is only interested in the *AND* events, where the probability can be estimated as^41^

3 $$\begin{aligned} P(sep_{yr}> q_{sep}, oct_{yr} > q_{oct}) = 1 - F_{sep}(q_{sep}) - F_{oct}(q_{oct}) + C(F_{sep}(q_{sep}), F_{oct}(q_{oct})), \end{aligned}$$

and the return period can be computed as

4 $$\begin{aligned} RP_{sep,oct_{yr}} = \frac{1}{P(sep_{yr}> q_{sep}, oct_{yr} > q_{oct})}, \end{aligned}$$

where $RP_{sep,oct_{yr}}$ is the return period of the compound event of September and October.

In this study, the joint return period of September and October is computed by three different pairs of quantiles to evaluate different aspects. First, the non-exceedance probability of the residuals for September and October are used as paired input to the calculation of the joint probability. This gives us information about the temporal evolution of compound extremes, without considering climate change, or interannual variability. Second, we want to compute the probability of the extreme event 2023, occurring in each of the last 249 years. Therefore, the fitted trend is subtracted from the anomalies of 2023 for each year, which can then be used as pairs of quantiles for each year. Finally, the occurrence probability of an event, that was at least average separately for each month in the last 10 years (2014–2023) is computed. Here, the average mean monthly anomalies for the years 2014 to 2023 is calculated for each month separately and subsequently the fitted trend is removed, which yields the final pairs of quantiles. This final analysis is not only performed for the compound events of September and August, but also for July and August.

### Supplementary Information

Supplementary Information.

## Data Availability

Data and code used for this study is available in a Zenodo publication: https://doi.org/10.5281/zenodo.10103330.
