# Supplementary material for: Climate change contribution to the 2023 autumn temperature records in Vienna
Source: Sci Rep. 2024 Feb 20;14:4213. doi: 10.1038/s41598-024-54822-2 (PMC10879489; doi:10.1038/s41598-024-54822-2)
Supplement: Supplementary file 1 — Supplementary Information. [file 41598_2024_54822_MOESM1_ESM.pdf]

Supplementary material

Modeling the relationship between monthly temperature in Vienna and the ENSO index

One issue of this study, is that current global temperature extremes are related to anomalies in the sea surface temperature, specifically the strong El Nino event, but the effect of El Nino on local climates is diverse. To exclude a possible impact of the current El Nino event, the monthly temperature anomalies of September and October in Vienna were regressed by the multivariate ENSO index.

This analysis is only performed for the period from 1980 to 2022 - as ENSO index values are very robust due to the availability of satellite derived SST and 43 year of data are sufficient for this analyses. Each monthly ENSO value, and averaged monthly values of 3,6 and 12 months were used as predictor variables, resulting in a total of 48 predictors. Due to the high dimension of the model matrix, a Lasso model[1,2] was applied. Lasso can shrink coefficients of a linear model to zero by penalizing the regression coefficient by a L1-norm. If no predictor variable has a merit to the model, this would lead to a linear model with only an intercept. The Lasso model is built not just once, but a 1000 times on different bootstrap samples with a size of 30 years. For evaluating the influence of the ENSO index, the number of times is counted where the Lasso model produces a fit where any of the predictor variables is included.

The results show that for the monthly temperature anomalies in Vienna only in 1.6 % of the cases (the model is repeated a 1000 times) in September, and in 6.9 % of the time in October any of the indices is added to the model. As there is only a weak sign that El Nino matters for the local climate in Vienna, we neglect the impact of El Nino in our modeling approach, although highly non-linear effects[3] or weak interactions between the predictors were not considered.

References

1. Tibshirani, R. Regression shrinkage and selection via the lasso. *J. Royal Stat. Soc. Ser. B (Methodological)* **58**, 267–288, DOI: https://doi.org/10.1111/j.2517-6161.1996.tb02080.x (1996).

2. Friedman, J., Hastie, T. & Tibshirani, R. Regularization paths for generalized linear models via coordinate descent. *J. Stat. Softw.* **33**, 1–22, DOI: 10.18637/jss.v033.i01 (2010).

3. Brönnimann, S. Impact of el niño–southern oscillation on european climate. *Rev. Geophys.* **45**, DOI: https://doi.org/10.1029/2006RG000199 (2007).

Supplementary Figures

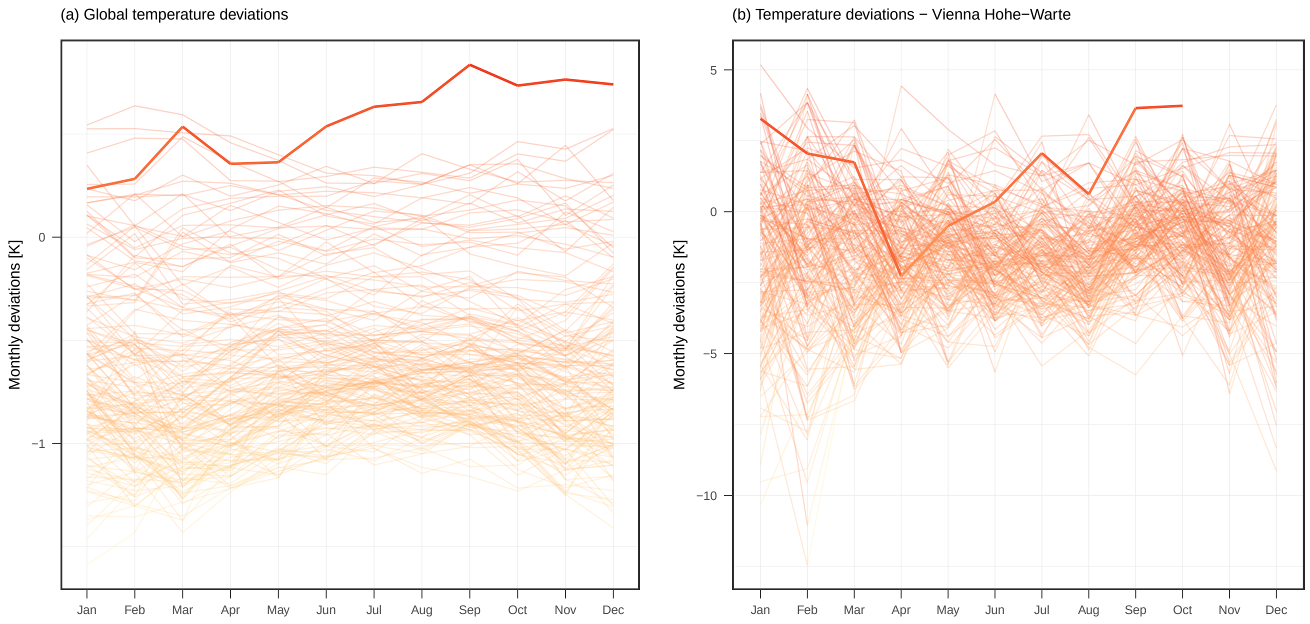

Supplementary Figure 1. Global vs. local temperature Presentation of the annual cycle from 1850 to 2023 for each year separately for global average temperature and the station Vienna Hohe-Warte. Deviations are computed by subtracting the climate reference period of 1991-2020. The thick line represents 2023 in both panels.

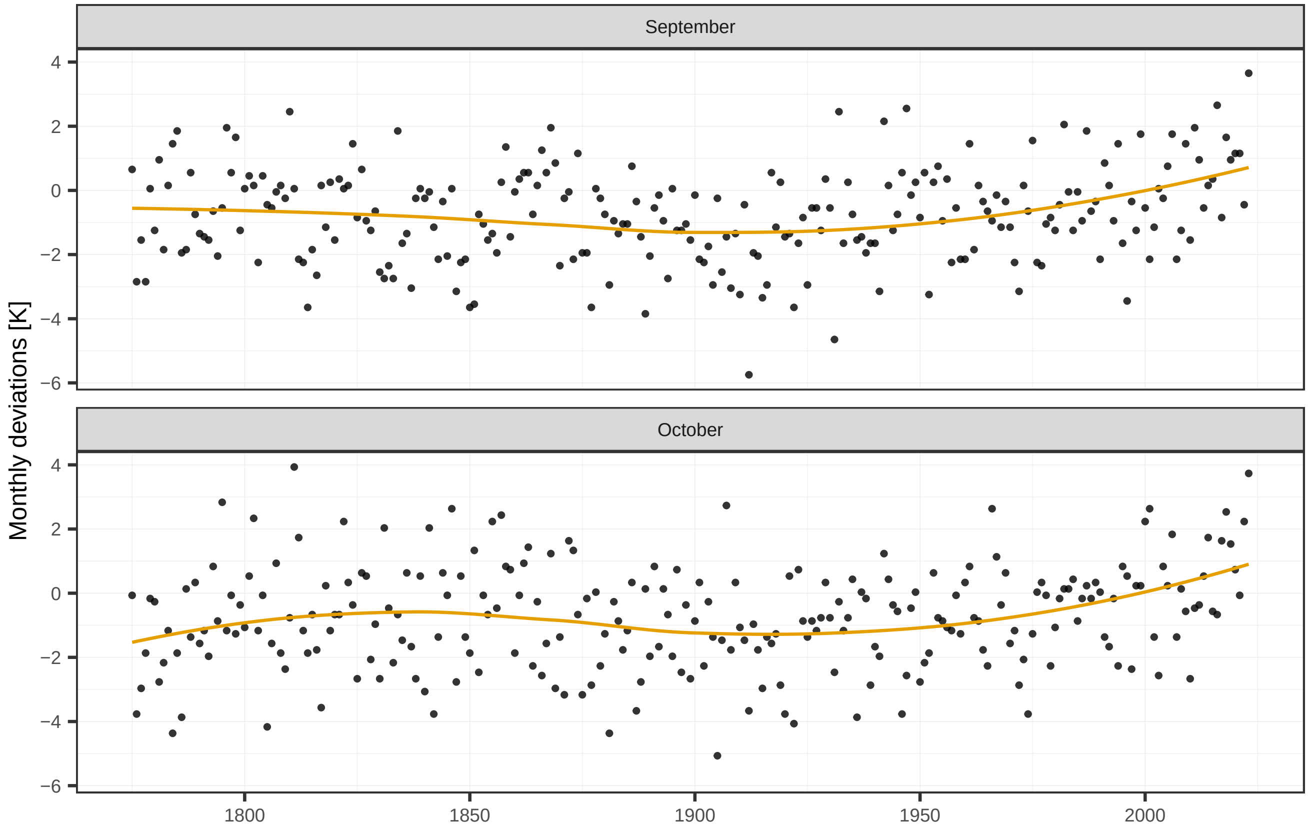

Supplementary Figure 2. **Loess-model** The monthly deviations for September and October are plotted as points for the station Vienna Hohe-Warte for the period of 1775 to 2023. The line plot is the fitted trend by a Loess-model for each of the months separately.

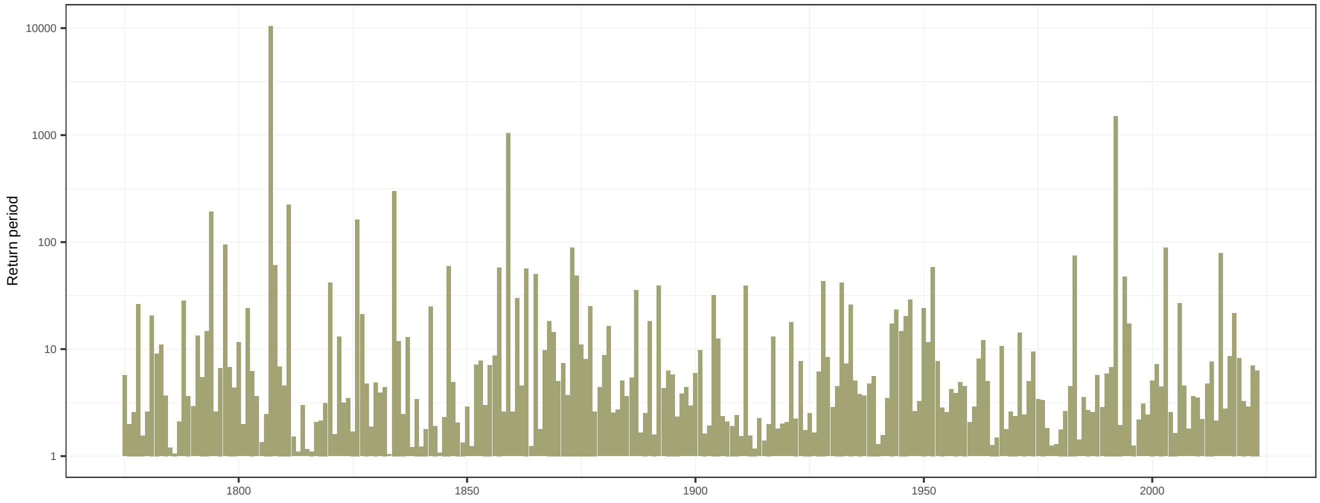

Supplementary Figure 3. **Compound extremes for July and August** Return periods of the compound effect of July and August temperature records, without considering climate change.
